# Léiomyosarcome gastrique simulant une tumeur du hile splénique: à propos d'un cas

**DOI:** 10.11604/pamj.2015.21.263.7301

**Published:** 2015-08-07

**Authors:** Mohamed Tarchouli, Ahmed Bounaim, Moulay Brahim Ratbi, Mohamed Said Belhamidi, Abdelhak Bensal, Badr Aitidir, Adil Boudhas, Abdelmounaim Ait Ali, Khalid Sair

**Affiliations:** 1 Service de Chirurgie Viscérale I, Hôpital Militaire d'Instruction Mohammed V, Université Mohammed V, Faculté de Médecine et de Pharmacie, Rabat, Maroc; 2 Service d'Anatomie Pathologique, Hôpital Militaire d'Instruction Mohammed V, Université Mohammed V, Faculté de Médecine et de Pharmacie, Rabat, Maroc

**Keywords:** Léiomyosarcome, estomac, masse abdominale, leiomyosarcoma, stomach, abdominal mass

## Abstract

Depuis la découverte de leur phénotype particulier, les tumeurs stromales gastro-intestinales représentent les tumeurs mésenchymateuses les plus fréquentes du tractus digestif et ne sont plus confondues avec les vrais léiomyosarcomes gastriques devenant ainsi exceptionnellement rencontrés dans la pratique médicale. Nous rapportons le cas d'une jeune femme de 32 ans admise pour une masse douloureuse de l'hypochondre gauche et chez qui le bilan radiologique objectivait une volumineuse tumeur occupant le hile splénique. Une résection monobloc emportant la masse, la rate, le grand épiploon et une collerette de la paroi gastrique a été effectuée et l'examen histologique a confirmé le diagnostic d'un léiomyosarcome gastrique. Il est extrêmement important de différencier les autres tumeurs mésenchymateuses du tractus digestif des léiomyosarcomes gastriques dont l'exérèse chirurgicale complète reste, jusqu’à présent, le seul traitement à visée curative.

## Introduction

Depuis la découverte du phénotype particulier des tumeurs stromalesgastrointestinales (GIST) avec l'expression fréquente du récepteur CD34 et celle quasi constante de la protéine c-Kit, ces tumeurs sont réellement individualisées et actuellement clairement distinguées des autres tumeurs mésenchymateuses digestives telles les léiomyomes, léiomyosarcomes, neurofibromes ou schwannomes. Les léiomyosarcomes (LMS) gastriques sont des tumeurs développées aux dépens des fibres musculaires lisses de la paroi gastrique devenant actuellement extrêmement rare avec le développement du concept des tumeurs stromales gastrointestinales. Selon la littérature, ils représentent seulement 1-3% de toutes les tumeurs malignes de l'estomac [1, 2]. Ils touchent généralement les adultes dans la cinquantaine sans aucune prédominance de sexe. Leur évolution silencieuse et le développement tumoral exophytique donnent une symptomatologie pauvre expliquant leur découverte tardive à l'occasion d'un saignement occulte ou d'une masse abdominale. Nous rapportons le cas d'un léiomyosarcome à développement exophytique révélé par une masse abdominale chez une jeune patiente de 32 ans. Notre objectif étant de rappeler aux différents cliniciens les principales caractéristiques diagnostiques et thérapeutiques de cette pathologie assez particulière.

## Patient et observation

**Figure 1:**
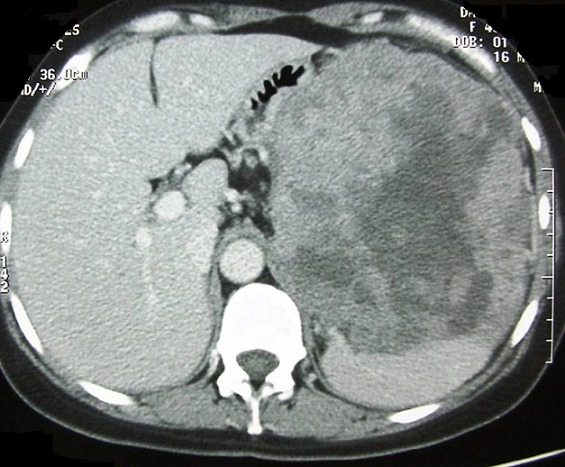
Coupe axiale de la TDM abdominale montrant une volumineuse masse occupant le hile splénique à contenu hétérogène renfermant quelques zones de nécroses centrales

**Figure 2:**
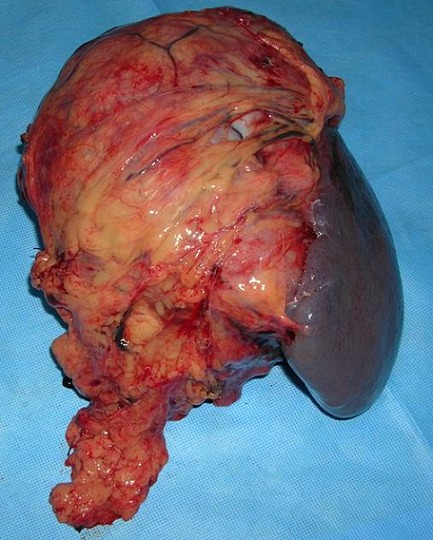
Pièce de résection chirurgicale emportant la masse, la rate, le grand épiploon, et une partie de la paroi gastrique

**Figure 3:**
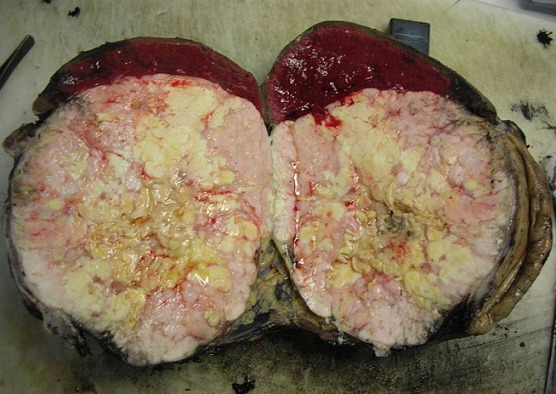
Aspect macroscopique de la masse adhérente à la paroi gastrique mais qui refoule le parenchyme splénique: a noter le contenu hétérogène et les zones de nécrose centrales

**Figure 4:**
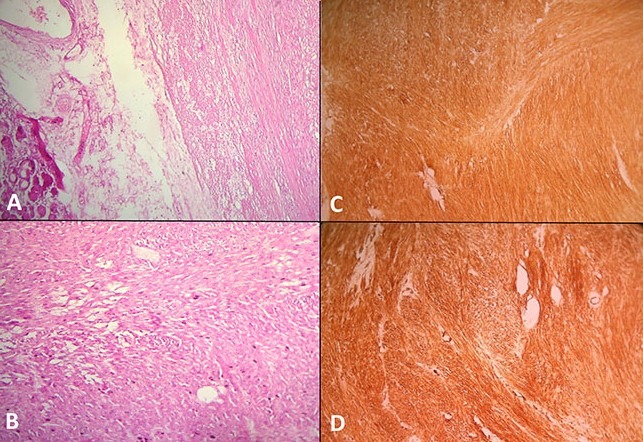
Etude histologique de la pièce opératoire montrant une prolifération tumorale faite de faisceaux anarchiquement enchevêtrés de cellules fusiformes (A) HEx4; (B) HEx20; (C) avec une expression diffuse à l'immunohistochimie de l'actine muscle lisse x20; (D) de la desmine x20

Nous rapportons le cas d'une jeune femme de 32 ans sans antécédents particuliers et jamais opérée admise pour des douleurs de l'hypochondre gauche à type de pesanteur évoluant depuis plus de 10 mois. Ces douleurs d'installation progressive sont accompagnées de vomissements intermittents et d'un amaigrissement non chiffré mais sans fièvre ni singes d'hémorragie digestive. L'examen clinique trouvait une sensibilité de l'hypochondre gauche avec une masse palpable, de consistance ferme mobile par rapport au plan superficiel et fixe par rapport au plan profond. Il n'y avait pas d'hépatomégalie et les aires ganglionnaires étaient libres. L’échographie abdominale avait objectivé une masse de consistance tissulaire à contenu hétérogène localisée au niveau du hile splénique. Une TDM abdominale a été ainsi réalisée pour mieux caractériser cette masse. Elle a montré une volumineuse masse de 20 cm de grand axe d'allure tissulaire adhérente à la rate et au grand épiploon et refoulant la face postérieure de l'antre gastrique sans l'envahir. Elle se rehausse de façon hétérogène après injection du produit de contraste laissant apparaitre quelques zones de nécroses centrales (Figure 1). En plus, il n'y avait pas d’épaississement de la paroi gastrique ni de signes de nodules hépatiques ou d'adénopathies profondes. La fibroscopie œsogastroduodénale avec biopsies n'avait pas montré d'anomalies remarquables alors que les marqueurs tumoraux incluant l'alpha-fœto-protéine, l´antigène carcino-embryonnaire et le CA-19.9 étaient dans les limites normales. Une laparotomie exploratrice a été ainsi indiquée permettant de réaliser, après libération des adhérences, une exérèse monobloc emportant la masse, la rate, le grand épiploon et une collerette de la paroi gastrique (Figure 2). L'exploration de la cavité abdominale n'avait pas trouvé de lésions hépatiques ni de carcinose péritonéale. Les suites opératoires étaient simples et la patiente a été sortie 7 jours plus tard. L'examen histologique de la pièce opératoire a découvert une tumeur mesurant 21x14x12 cm qui comble le hile de la rate en refoulant son parenchyme et adhère intimement à la paroi gastrique avec des foyers nécrotiques prédominant au centre (Figure 3). L’étude microscopique était en faveur d'une prolifération sarcomateuse faite de cellules fusiformes avec des limites de résection gastriques saines et une rate non envahie par le processus tumoral. L’étude immunohistochimique a révélé une positivité intense et diffuse des cellules tumorales à l'actine muscle lisse et à la desmine alors que la protéine S100, le CD117 et le CD34 étaient clairement négatifs (Figure 4). Le diagnostic d'un léiomyosarcome d'origine gastrique a été retenu mais aucun traitement adjuvant n'a été indiqué chez cette patiente décédée 2 ans plus tard dans un tableau de métastases pulmonaires.

## Discussion

Les léiomyosarcomes sont des tumeurs mésenchymateuses développées de façon ubiquitaire dans le tissu de soutien des muscles lisses avec un siège de prédilection au niveau de l'utérus et le tractus gastrointestinal en particulier l'estomac et l'intestin grêle. La plupart des léiomyosarcomes du tube digestif se situe dans l'estomac où ils représentent 1à 3% des tumeurs gastriques malignes et environ 20 à 30% de l´ensemble des sarcomes gastriques [3, 4]. Cependant, depuis le développement du concept des tumeurs stromales gastro-intestinales, ces dernières représentent les tumeurs mésenchymateuses les plus fréquentes du tractus digestif. Ainsi, les léiomyosarcomes gastriques sont devenus extrêmement rares de nos jours. La pathogénie de ces tumeurs n'est pas encore clairement élucidée. Les léiomyomes présentent le risque théorique de dégénérescence mais sans aucune preuve histologique alors que des associations lésionnelles ont été décrites dans le cadre de la triade de Carney comportant un léiomyosarcome gastrique, un chondrome pulmonaire et un paragangliome extra-surrénalien fonctionnel [5]. Les léiomyosarcomes gastriques touchent les adultes dans la cinquantaine, mais ils sont rares chez les jeunes adultes comme c'est le cas de notre patiente. Les hommes et les femmes partagent les mêmes risques de contracter cette maladie.

Le retard diagnostique souvent constaté est dû au manque de spécificité des symptômes révélateurs et du développement souvent exogastrique de ces tumeurs comme c'est le cas dans notre observation [5]. Les lésions sont généralement localisées au niveau de la paroi postérieure du corps et de la grande courbure gastriques [3]. La symptomatologie clinique dépend ainsi de la taille de la lésion, de sa vitesse de croissance et de son mode de développement endo ou exoluminal. Les signes révélateurs sont représentés essentiellement par l´hémorragie digestive, la douleur, la masse abdominale, la perte de poids, et la fièvre [5, 6]. Les complications révélatrices telles que l'hémorragie intrapéritonéale, la péritonite aigue ou la sténose pylorique sont rarement rapportés. En dehors de la découverte d'une masse abdominale, l'examen physique est le plus souvent normal. La fibroscopie gastrique est peu contributive au diagnostic car ces lésions à développement sous muqueux sont souvent à prédominance exophytique. La fibroscopie peut montrer un aspect normal ou simplement une voussure endo-luminale provoquée par un processus expansif extrinsèque. Les biopsies perendoscopiques sont généralement superficielles et négatives [2]. L’écho-endoscopie est l'examen de référence qui permet d'affirme le caractère intrapariétal de la lésion ou au contraire son origine extrinsèque avec une sensibilité avoisinant les 97% [7]. Elle permet aussi d’étudier les rapports de la tumeur avec les organes de voisinage, de juger le degré d'envahissement et de préciser l'existence, quoique rare, de ganglions loco-régionaux [8]. Le transit œsogastroduodénal reste un examen important pour le diagnostic. Il révèle souvent une image lacunaire évocatrice de cette tumeur sous-muqueuse. La TDM abdominale sans puis avec injection du produit de contraste et ingestion d'eau ou de la Gastrograffine sur la table d'examen est un outil intéressant pour analyser ces tumeurs surtout les lésions de plus de 10 cm et pour rechercher les métastases surtout hépatiques [5, 7]. Les images sans injection permettent de visualiser les hémorragies intra-tumorales et les rares calcifications endo-lésionnelles. Au temps artériel, ces tumeurs se rehaussent de façon hétérogène et fugace en raison de leur hypervascularisation avec l'apparition des images hypodenses centrales de nécrose tumorale. Les léiomyosarcomes gastriques à développement exoluminalrefoulent les organes intra et/ou rétro péritonéaux et peuvent prêter confusion avec des lésions des organes de voisinage.

Chez notre patiente, la tumeur à développement exophytique a été confondue avec une tumeur du hile splénique. Les métastases hépatiques de contiguïté sont fréquentes constituant un élément de mauvais pronostic mais les métastases ganglionnaires sont exceptionnelles [7]. Le diagnostic d'un léiomyosarcome gastrique est difficile en préopératoire, il se fait en général à l'examen anatomopathologique d'une pièce de résection chirurgicale. Histologiquement les léiomyosarcomes sont constitués de cellules fusiformes avec un cytoplasme éosinophile, des noyaux centraux d´aspect boudiné et une activité mitotique remarquablement élevée. Ils restent toutefois largement confondus aux tumeurs stromalesgastrointestinales du moins en endoscopie et en imagerie. Le diagnostic de certitude est fait grâce à l’étude immunohistochimique qui montre une expression diffuse des marqueurs musculaires principalement l'actine muscle lisse associée à l'absence de l'expression du CD117 et le CD34 [1, 9]. Ce profil histologique qui correspond parfaitement à notre observation peut poser le problème de diagnostic différentiel avec les autres tumeurs mésenchymateuses à cellules fusiformes comme le léiomyome, le léiomyoblastome et les schwannomes. L'exérèse chirurgicale complète de la tumeur avec des marges saines reste le traitement standard des léiomoysarcomes gastriques. Une gastrectomie atypique (résection cunéiforme) avec des marges de sécurité minimales de 2 cm peut être suffisante, mais une gastrectomie subtotale s'impose dans le cas d'une volumineuse tumeur [10]. Parfois une résection d'un organe de voisinage s'avère nécessaire pour assurer l'excision complète du tissu tumoral. Par ailleurs le curage ganglionnaire n'est pas systématique car il n'influence pas de façon significative la survie des malades [2]. Concernant les thérapeutiques adjuvantes, les léiomyosarcomes sont connus résistants à la radiothérapie et à la chimiothérapie [2]. Le pronostic des léiomyosarcomes dépend des métastases viscérales synchrones, de la taille de la tumeur (supérieure à 6 cm de mauvais pronostic), du grade histologique, et de l'infiltration pariétale du reste de l'estomac [2, 7]. Le taux de survie à 5 ans des patients atteints de léiomyosarcomes est de 22% [2, 7].

## Conclusion

Les vrais léiomyosarcomes gastriques sont devenus extrêmement rares avec les avancées de l'immunohistochimie et de la biologie moléculaire qui permettent de les distinguer, d'une façon claire, des tumeurs stromales gastro-intestinales représentant actuellement la majorité des tumeurs mésenchymateuses intestinales. Une étude spéciale concernant cette pathologie semble irréalisable vu sa rareté. Ainsi il serait intéressant de continuer à étudier d'autres cas de léiomyosarcomes gastriques pour une compréhension plus profonde de cette pathologie assez particulière.
